# Bone mineral density in adults with arthrogryposis multiplex congenita: a retrospective cohort analysis

**DOI:** 10.1038/s41598-024-58083-x

**Published:** 2024-04-08

**Authors:** X. Romand, R. Gastaldi, D. Pérennou, A. Baillet, K. Dieterich

**Affiliations:** 1 T-RAIG, TIMC, CNRS, UMR 5525, University of Grenoble Alpes, Grenoble-Alpes University Hospital, 38000 Grenoble, France; 2 Rheumatology Department, Grenoble-Alpes University Hospital, Grenoble, France; 3 Department of PMR, University of Grenoble Alpes, UMR CNRS 5105 LPNC, Grenoble-Alpes University Hospital (South Site), Cs 10217, 38043 Grenoble cedex 9, France; 4 Medical Genetics, Institute of Advanced Biosciences, University of Grenoble Alpes, Inserm, U1209, CHU Grenoble Alpes, 38000 Grenoble, France

**Keywords:** Arthrogryposis, Amyoplasia, Bone mineral density, Fracture, Vitamin D deficiency, Medical research, Rheumatology

## Abstract

The primary objective of this study was to evaluate the prevalence of low femoral and lumbar spine bone mineral density (BMD) in adults with arthrogryposis multiplex congenita (AMC). We performed a retrospective cohort analysis of adults with AMC who were enrolled in the French Reference Center for AMC and in the Pediatric and Adult Registry for Arthrogryposis (PARART, NCT05673265). Patients who had undergone dual-energy X-ray absorptiometry (DXA) and/or vitamin D testing were included in the analysis. Fifty-one patients (mean age, 32.9 ± 12.6 years) were included; 46 had undergone DXA. Thirty-two (32/51, 62.7%) patients had Amyoplasia, and 19 (19/51, 37.3%) had other types of AMC (18 distal arthrogryposis, 1 Larsen). Six patients (6/42, 14.3%) had a lumbar BMD Z score less than − 2. The mean lumbar spine Z score (− 0.03 ± 1.6) was not significantly lower than the expected BMD Z score in the general population. Nine (9/40, 22.5%) and 10 (10/40, 25.0%) patients had femoral neck and total hip BMD Z scores less than − 2, respectively. The mean femoral neck (− 1.1 ± 1.1) and total hip (− 1.2 ± 1.2) BMD Z scores in patients with AMC were significantly lower than expected in the general population (p < 0.001). Femoral neck BMD correlated with height (rs = 0.39, p = 0.01), age (rs = − 0.315, p = 0.48); total hip BMD correlated with height (rs = 0.331, p = 0.04) and calcium levels (rs = 0.41, p = 0.04). Twenty-five patients (25/51, 49.0%) reported 39 fractures. Thirty-one (31/36, 86.1%) patients had 25-hydroxyvitamin D levels less than 75 nmol/l, and 6 (6/36, 16.7%) had 25-hydroxyvitamin D levels less than 75 nmol/l. Adults with AMC had lower hip BMD than expected for their age, and they more frequently showed vitamin D insufficiency. Screening for low BMD by DXA and adding vitamin D supplementation when vitamin D status is insufficient should be considered in adults with AMC, especially if there is a history of falls or fractures.

## Introduction

Arthrogryposis multiplex congenita (AMC) is a heterogeneous group of congenital conditions characterized by joint contracture in at least two parts of the body^1^. AMC classification can be challenging. The most useful distinction in clinical practice is among (i) Amyoplasia, a sporadic anterior horn cell disease characterized by selective atrophy or absence of limb girdle muscles and upper and lower limb muscles; (ii) distal arthrogryposis; and (iii) nonspecified AMC^2^. The causes of fetal hypokinesia or akinesia leading to the development of AMC are variable and include gene mutation, bacterial or viral infection, teratogenic factors and mechanical restrictions of intrauterine movement^3,4^.

Mechanical loading is an important factor in the regulation of bone growth and metabolism through its involvement in the proliferation and differentiation of osteoblasts^5^. Newborns with congenital neuromuscular diseases that restrict fetal movement and decrease mechanical stimulation during intrauterine development, as observed in AMC, develop osteopenia and low bone cortical thickness^6^. Hypomineralization and hypoechogenicity of the long bones in the third trimester of pregnancy were described in a case report^7^. Ten percent of newborns with AMC experience long bone fractures during delivery or the perinatal period^8^. Patients with AMC have muscle weakness and joint contractures, poor mobility and mechanical arthropathy, which are factors known to increase the risk of falls that can lead to fracture^9–11^. Fifty-eight percent of adult patients with AMC report having experienced multiple falls, which is a greater percentage than in the general population^12^. Nevertheless, few studies have evaluated bone mineral density (BMD) in patients with AMC or compared BMD among specific AMC subgroups.

Two previous cross-sectional studies reported low lumbar spine BMD in children and adolescents with AMC^13,14^. Dahan-Oliel et al*.* proposed that a smaller body size that affects bone geometry rather than a specific BMD deficit could explain their findings; this led them to not recommend regular bone densitometry monitoring, except in specific cases, such as patients with a history of fractures^13^.

To our knowledge, no previous studies have assessed BMD by dual-energy X-ray absorptiometry (DXA) in adult patients with AMC to determine whether the low BMD observed in childhood persists to low BMD in adulthood.

A low BMD may be explained by a lack of mechanical loading from intrauterine development into adulthood. The primary study objective was to evaluate the prevalence of low lumbar spine and hip BMD in a cohort of adults with AMC. The secondary objectives were to assess the prevalence of 25-hydroxyvitamin D (25-OHD) deficiency, disorders of calcium and phosphate metabolism, and fractures and to identify factors associated with low BMD in adult patients with AMC. We hypothesized that the low BMD observed in childhood persists into adulthood in patients with AMC.

## Methods

### Patients and study design

We performed a retrospective cohort analysis of adult patients with AMC enrolled in the French Reference Center for AMC and in the Pediatric and Adult Registry for Arthrogryposis from 2010 to 2020 (Genetics, Neurorehabilitation and Rheumatology Departments, University Hospital Grenoble-Alpes, France)^11^. Patients with AMC (i) who were older than 16 years (ii) with a confirmed diagnosis of AMC fulfilling the criteria of the international AMC classification^1^ (iii) and who had undergone DXA and/or vitamin D assays were included. All patients were evaluated by a clinical geneticist trained in AMC and neuromuscular disorders (KD). For 5 days, the patients underwent a multidisciplinary management program in which all requisite investigations were carried out (biology, functional tests, DXA analysis, etc.). Patients were divided into two subgroups, (i) the Amyoplasia subgroup and (ii) the other AMC subgroup, according to clinical signs and molecular diagnosis. The reporting of the study follows the Strengthening the Reporting of Observational Studies in Epidemiology guidelines.

### Ethics

This study was performed in compliance with the Declaration of Helsinki. In accordance with French data protection law (JORF Official Journal, May 10, 2017), all participants provided informed consent; the study did not require approval from an ethics committee (Decree n° 2019-536, May 29, 2019). All data used in this study were obtained from the Pediatric and Adult Registry for patients with ARThrogryposis multiplex congenita (PARART). This noninterventional study was declared to the research department of the University Hospital Grenoble-Alpes and registered on Clinicaltrials.gov (NCT 05673265).

### DXA analysis

Lumbar spine (L1-L4), femoral neck and total hip BMD were measured by DXA in the University Hospital Grenoble-Alpes Radiology Department, which specializes in osteoarticular disease. All BMD values were obtained on the same densitometer (Lunar iDXA, GE Healthcare). Lumbar spine, right and left femoral neck and total hip BMD (g/cm^2^) were transformed to age- and sex-specific Z scores according to the manufacturer’s reference data. As our population was predominantly young (premenopausal women and men under 50 years), we chose to use the Z score, in accordance with the 2023 Official Positions for Adults of the International Society of Clinical Densitometry (ISCD)^15^. A Z score of − 2.0 or less indicated that the BMD was below the expected range for individuals of that age. The Z score is the number of standard deviations from the mean BMD of a healthy population of the same age and sex. Quality control for instrumentation was performed daily, using a spine phantom provided by the manufacturer, prior to any measurement. Lumbar spine, femoral neck and total hip BMD and Z score values were collected from the patients’ medical records by one of the investigators (XR). The quality of BMD analysis was graded by two independent investigators (XR, RG) as perfect, not perfect but interpretable, or uninterpretable. If the quality score was equivalent between the left and right femurs, the side with the lower femoral neck BMD and Z score was selected. Lumbar and femoral BMD and Z scores from DXA analyses considered uninterpretable were excluded from the statistical analysis.

### 25-OHD, calcium and phosphate levels

25-Hydroxyvitamin D (OH-D) levels were assessed in plasma via liquid chromatography-tandem mass spectrometry on a Dionex Ultimate 3000RS HPLC (Thermo Fisher Scientific, Waltham, MA, USA) coupled to an ABSciex 4000 triple quadrupole mass spectrometer (ABSciex, Foster City, CA, USA)^16^. The cutoff values used for the classification of vitamin D status were as follows: < 25 nmol/l, severe deficiency; 25–49 nmol/l, deficiency; 50–74 nmol/l, insufficiency; and ≥ 75 nmol/l, normal vitamin D status.

Calcium and phosphate levels were measured via a colorimetric assay (Vista – Siemens). Calcium levels < 2.12 mmol/l and > 2.60 mmol/l indicated hypocalcemia and hypercalcemia, respectively. Phosphate levels < 0.80 mmol/l and > 1.45 mmol/l indicated hypophosphatemia and hyperphosphatemia, respectively. Calcium, phosphate and 25-OHD levels were collected from the patients’ medical records by one of the investigators (XR).

### Clinical study parameters

All participants underwent a multidisciplinary evaluation involving physicians (a geneticist, physical medicine and rehabilitation physician, pneumologist and rheumatologist), a psychologist, physiotherapists, occupational and speech therapists and nurses. Lifetime fracture history, height and weight were collected from the medical records of the 5-day multidisciplinary assessment program by one of the investigators (XR). Functional exercise capacity was assessed via the 6-minute walk test (6MWT)^17,18^. The patient walked as far as possible in 6 min, safely and without running. Encouragement was standardized. Every minute, the healthcare professional announced the time remaining^11^. Limited ambulation was defined as an inability to walk or a 6MWT distance < 130 m (20% of the mean 6MWT distance for healthy adults aged 20–50 years^19^). Functional status in daily living was evaluated with the Functional Independence Measure (FIM)^20^. The FIM is an 18-item ordinal scale (score range, 18–126)^20^. Thirteen items assess motor abilities, and 5 assess cognitive abilities (scores range from 1 point per item if total assistance is required to 7 points per item for complete independence).

### Statistical analysis

Mann‒Whitney tests and *t* tests were used to compare two groups with nonnormally and normally distributed data, respectively. The Shapiro–Wilk test was used to test for a normal distribution. Variables are expressed as the mean ± standard deviation (SD) or median [first quartile (Q1)–third quartile (Q3)].

For categorical data, the chi-square test or Fisher’s exact test was used to compare two groups. Categorical data are expressed as numbers and percentages.

To test whether the mean Z scores were significantly different from zero (i.e. the mean result expected in the general population), we used a one-sample *t* test. Correlations between lumbar spine, femoral neck and total hip BMD and age, height, weight, body mass index (BMI), calcium level, phosphate level, 25-OHD level, 6MWT distance and total FIM score were assessed via Spearman rank correlation tests (r_s_). Age, sex, type of AMC, height, weight, BMI, history of fracture, calcium level, phosphate level, 25-OHD, 6MWT distance, total FIM score and limited ambulation were evaluated as potential factors associated with lumbar or femoral neck BMD Z scores lower than − 2 using univariate logistic regression analysis. Patients with missing data were excluded from the statistical analysis; missing data are reported in the Results tables. A p value < 0.05 was considered to indicate significance. Statistical analyses were performed using Jamovi (version 1.6.23) and R +  + software (version 1.5.07).

## Results

### Patient characteristics

**Figure 1:**
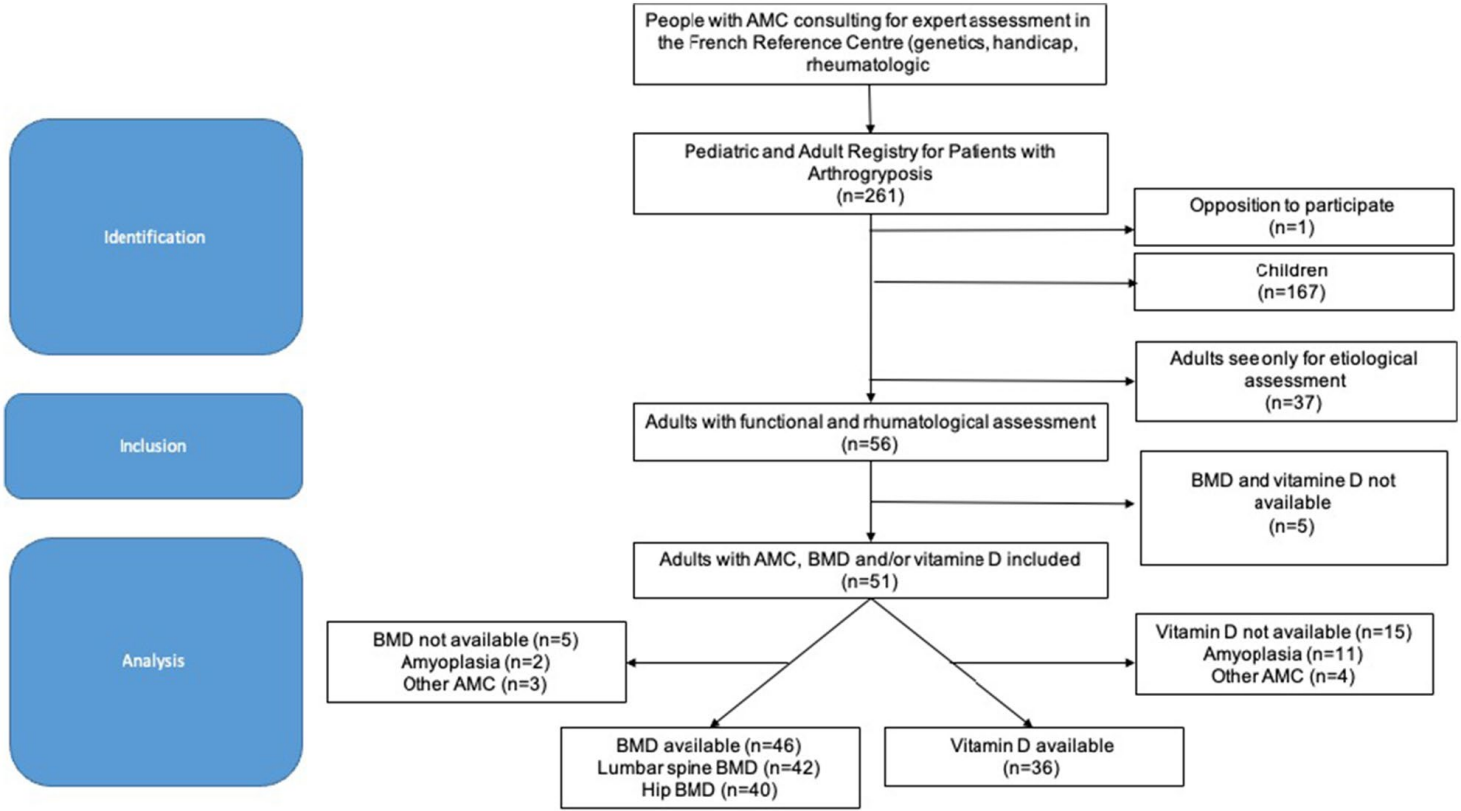
Flowchart. *BMD* bone mineral density, *AMC* arthrogryposis multiplex congenital.

Fifty-six patients with AMC were assessed (Fig. 1). Fifty-one (51/56, 91.1%) patients had undergone a 25-OHD assay and/or DXA and were included in the retrospective analysis. According to the clinical presentation and molecular diagnosis, 32 of the 51 patients (32/51, 62.7%) were classified as having Amyoplasia, and 19 (19/51, 37.2%) were classified as having other types of AMC: 16 cases of distal arthrogryposes due to pathogenic variants in *PIEZO2* (n = 4), *ECEL1* (n = 3), *TNNI2* (n = 3), *TPM2* (n = 2), *MYH3* (n = 1), *FBN2* (n = 1), *CHRNG* (n = 1), and *TTN* (n = 1); 1 case of *FLNB*; 1 case of *TRPV4*; and 1 case of *ZC4H2*. The mean age was 32.9 ± 12.6 years. Thirty-four patients were female (34/51, 66.7%). Height, weight, BMI and age were comparable between the Amyoplasia and other types of AMC groups. Baseline characteristics are shown in Table 1. There were no significant differences between females and males (Supplementary Table 1) or between patients who were included in the study and those who were excluded (data not shown).

**Table 1:** Characteristics of adult patients with arthrogryposis multiplex congenita.

Patient characteristics	n	All patients with AMC, n = 51	Amyoplasia, n = 32	Other types of AMC, n = 19	p value
Age (years) (median [Q1-Q3])	51	30 [25–41]	29.5 [25.3–42.5]	30 [25.0–35.0]	0.9
Female (n (%))	51	34 (66.7)	20 (62.5)	14 (73.7)	0.4
Disease classification	51				
Amyoplasia (n (%))		32 (62.7)	32 (100)	ND	ND
Distal arthrogryposis (n (%))		18 (35.3)	ND	18 (94.7)	ND
Other (Larsen) (n (%))		1 (2.0)	ND	1	ND
Height (cm) (mean (SD))	46	156 (9.9)	157 (7.9)	155 (12.7)	0.3
Weight (kg) (mean (SD))	46	57.9 (17.2)	60.9 (16.0)	52.9 (18.4)	0.1
BMI (kg/m^2^) (median [Q1-Q3])	46	21.9 [19.7–26.6]	22.3 [21.3–27.6]	20.4 [17.7–24.5]	0.2
History of fracture	51	25 (49.0)	15 (46.9)	10 (52.6)	0.7
Limited ambulation	51	14 (27.5)	9 (28.1)	5 (26.3)	0.9

*AMC* arthrogryposis multiplex congenita, *BMI* body mass index, *ND* no data. p values: Student’s t test, the Mann‒Whitney *U* test or the chi‒square test between patients with Amyoplasia and patients with other types of AMC. Q1: first quartile, Q3: third quartile.

### Lumbar spine, femoral neck and total hip BMD

**Figure 2:**
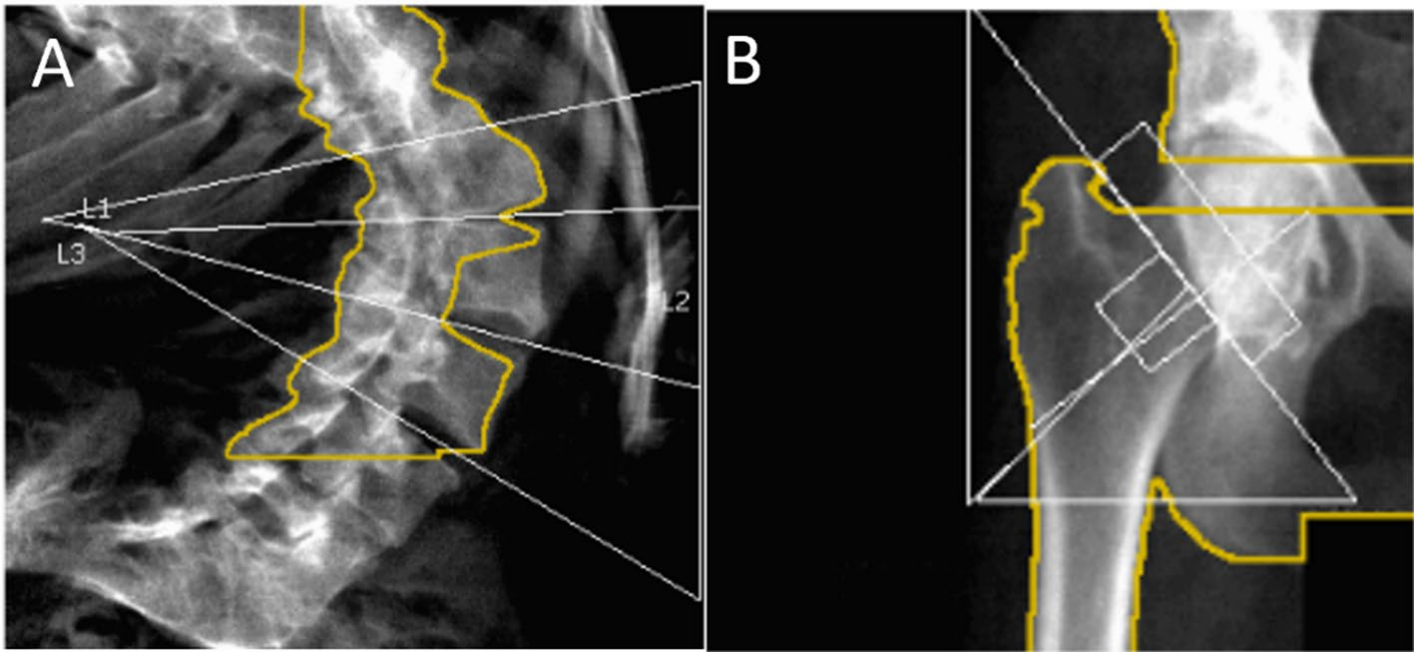
Arthrogryposis multiplex congenita and difficulties in measuring bone mineral density with dual-energy X-ray absorptiometry. The dual-energy X-ray absorptiometry results were uninterpretable for some arthrogryposis patients due to severe scoliosis (**A**) or hip joint contracture with mechanical arthropathy (**B**). These data were excluded from the analysis.

Forty-six (46/56, 82.1%) patients had undergone DXA at Grenoble Alpes University Hospital. There were no significant differences between patients who did not undergo DXA and patients who did undergo DXA (data not shown). Four patients had uninterpretable lumbar spine BMD results due to significant scoliosis or spinal fixation devices. Eighteen patients had uninterpretable femoral neck results, including 4 patients whose femoral neck BMD was uninterpretable on both sides because the region of interest could not be correctly positioned due to joint contractures (Fig. 2) and 2 patients whose femoral neck BMD was unavailable. None of the patients who underwent DXA had uninterpretable BMD results at all three sites (lumbar spine, right hip and left hip).

Lumbar spine DXA analyses revealed a mean BMD of 1.14 (± 0.21) g/cm^2^; 22 patients (22/42, 52.4%) had Z scores less than 0, and 6 patients (6/42, 14.3%) had Z scores less than -2. The mean BMD Z score for the lumbar spine was not significantly lower than zero. Individuals with other types of AMC had significantly lower Z scores than those with Amyoplasia (Table 2). The mean BMD Z score for the lumbar spine was not significantly lower than zero for patients with Amyoplasia or other types of AMC.

**Table 2:** Bone mineral density and vitamin D deficiency in adult patients with arthrogryposis multiplex congenita.

	n	All patients with AMC, n = 42	Amyoplasia, n = 27	Other types of AMC, n = 15	p value
Lumbar					
Bone mineral density (g/cm^2^) (mean (SD))	42	1.14 (0.21)	1.19 (0.18)	1.05 (0.24)	0.06
Z score (mean (SD))	42	− 0.03 (1.6)	0.35 (1.5)	− 0.720 (1.657)	**0.04**
Z score ≤ − 2 (n (%))	42	6 (14.3)	1 (3.7)	5 (33.3)	**0.01**
Z score < 0 (n (%))	42	22 (52.4)	11 (40.7)	11 (73.3)	**0.04**
Femoral neck					
Bone mineral density (g/cm^2^) (mean (SD))	40	0.832 (0.16)	0.853 (0.13)	0.788 (0.192)	0.2
Z score (mean (SD))	40	− 1.12 (1.1)	− 0.97 (1.0)	− 1.42 (1.29)	0.2
Z score ≤ − 2 (n (%))	40	9 (22.5)	5 (18.5)	4 (30.8)	0.3
Z score < 0 (n (%))	40	35 (85.0)	23 (85.2)	11 (84.6)	1.0
Total hip					
Bone mineral density (g/cm^2^) (mean (SD))	40	0.838 (0.160)	0.862 (0.126)	0.786 (0.212)	0.2
Z score (mean (SD))	40	− 1.24 (1.2)	− 1.07 (1.1)	− 1.58 (1.5)	0.2
Z score ≤ − 2 (n (%))	40	10 (25.0)	3 (11.1)	7 (46.7)	**0.003**
Z score < 0 (n (%))	40	33 (82.5)	22 (81.5)	11 (73.3)	0.8

*AMC* Arthrogryposis multiplex congenita. p values: Student’s *t* test or chi-square test between the Amyoplasia group and the other types of AMC group. Bold, significant p value (< 0.05).

The mean femoral neck and total hip BMD values were 0.832 (± 0.16) g/cm^2^ and 0.838 (± 0.16) g/cm^2^, respectively. There was no difference between right and left femoral neck BMD and total hip BMD (data not shown). Thirty-four patients (34/40, 85.0%) had a femoral neck Z score and 33 patients (33/40, 82.5%) had a total hip Z score less than 0. Nine (9/40, 22.5%) patients had a femoral neck Z score and 10 (10/40, 25.0%) had a total hip Z score less than -2. The mean femoral neck (-1.1 ± 1.1) and total hip (-1.2 ± 1.2) BMD Z scores were significantly lower than zero for all patients (p < 0.001) and for both subgroups (Amyoplasia and other types of AMC). The mean femoral neck and total hip BMD did not differ between the Amyoplasia subgroup and the other types of AMC subgroup (Table 2). The prevalence of total hip BMD Z scores below -2 was greater in the other types of AMC subgroup than in the Amyoplasia subgroup (7/15, 46.7% *vs*. 3/27, 11.1%, p = 0.003). A Z score of less than -2 at one site was observed in 30.4% (14/46) of patients with AMC.

Lumbar spine BMD correlated positively with height (rs = 0.36, p = 0.02), weight (rs = 0.37, p = 0.02) and BMI (rs = 0.33, p = 0.03) (Supplementary Table 2). Factors associated with a lumbar spine BMD Z score < -2 included shorter height (p = 0.02) and AMC type other than Amyoplasia (p = 0.04). Height and Amyoplasia were significantly associated with a lumbar spine BMD Z score < -2 (Table 3). Femoral neck BMD correlated with height (rs = 0.39, p = 0.01), age (rs = -0.315, p = 0.48); total hip BMD correlated with height (rs = 0.331, p = 0.04) and calcium levels (rs = 0.41, p = 0.04) (Supplementary Tables 3 and 4). Patients with limited mobility were not more likely to have a femoral neck BMD Z score < − 2 (4/9 (44.4%), p = 0.07). Height was significantly associated with a femoral neck BMD Z score < − 2 (Table 3). Other factors (age, sex, calcium level, hypocalcemia, phosphate level, hypophosphatemia, 25-OHD level, 25-OHD insufficiency, 25-OHD deficiency, weight, BMI, 6MWT distance, total FIM score, and limited ambulation) were not associated with a lumbar spine, femoral neck or total hip BMD Z score below − 2 or zero (Table 3).

**Table 3:** Factors related to lower bone mineral density in patients with AMC.

Factors	n	Univariate analysis, OR (95% CI)	p value
Lumbar spine BMD Z score < − 2			
Age	42	0.99 [0.91, 1.07]	0.75
Sex	42	0.88 [0.14, 5.54]	0.89
Not amyoplasia	**42**	**11.36 [1.18, 109.02]**	**0.04**
Height	**41**	**0.87 [0.77, 0.98]**	**0.02**
Weight	42	0.94 [0.89, 1.01]	0.08
BMI	42	0.93 [0.80, 1.08]	0.34
Calcium level	26	0.05 [5.43e–06, 471.28]	0.52
Phosphate level	25	2.81 [0.06, 133.69]	0.60
25-OHD level	28	0.97 [0.93, 1.02]	0.27
6MWT distance	41	0.99 [0.99, 1.00]	0.12
Limited ambulation	42	3.50 [0.59, 20.81]	0.17
Total FIM score	42	0.99 [0.94, 1.04]	0.74
History of fracture	42	1.79 [0.29, 11.03]	0.53
Neck BMD Z score < − 2			
Age	40	1.00 [0.94, 1.08]	0.82
Sex	40	5.05 [0.56, 45.6]	0.15
Not amyoplasia	40	1.96 [0.42, 9.00]	0.39
Height	40	**0.91 [0.84, 0.99]**	**0.04**
Weight	40	0.99 [0.95, 1.04]	0.81
BMI	40	1.03 [0.94, 1.14]	0.51
Calcium level	25	0.01 [3.52e–07, 61.9]	0.27
Phosphate level	24	9.31e–07 [1.56e–07, 5.55]	0.12
25-OHD level	27	0.99 [0.91, 1.09]	0.87
6MWT distance	39	1.00 [0.99, 1.00]	0.07
Limited ambulation	40	4.16 [0.82, 21.15]	0.09
Total FIM score	40	0.97 [0.92, 1.01]	0.16
History of fracture	40	0.75 [0.17, 3.33]	0.71

In bold: statistically significant (p value < 0.05).

*OR* odds ratio, *CI* confidence interval, *BMD* bone mineral density, *BMI* body mass index, *25-OHD* 25-hydroxyvitamin D, *6MWT* 6-min walk test, *FIM* functional independence measure.

### Fracture history and BMD

Twenty-five patients (25/51, 49.0%) reported 39 fractures (an average of 1.6 fractures per patient). Twenty-two (22/39, 56.4%) of the fractures involved the upper limb (7 humerus, 5 forearm, 3 elbow, 1 acromion, 1 scaphoid, 1 scapula and 4 unspecified locations), 41.0% (16/39) involved the lower limb (8 femur, 4 tibia, 1 calcaneum, 1 foot and 2 unspecified locations), and 2.6% (1/39) involved the pelvis. No cases of vertebral fracture were reported. A total of 42.1% (16/38) of fractures occurred after the age of 16. The proportion of fractures caused by low-level trauma equivalent to a fall from standing height or less could not be determined due to the lack of precise data in the medical records. The lumbar spine, femoral neck and total hip BMD and Z score did not differ according to the history of fracture.

### Blood 25-OHD, calcium and phosphate levels

Thirty-six (36/51, 70.6%), 33 (33/51, 64.7%) and 32 (32/51, 62.7%) patients underwent 25-OHD, calcium and phosphate assays, respectively. Patients who had undergone vitamin D assays had a lower weight (mean 53.58 ± 14.2 kg vs. 66.93 ± 19.7 kg, p = 0.012).

The mean 25-OHD was 48.5 ± 24.3 nmol/l. Thirty-one patients (31/36, 86.1%) had a 25-OHD below the normal value of 75 nmol/l.

Ten patients (10/36, 27.8%) had 25-OHD insufficiency (50–74 nmol/l), 15 (15/36, 41.7%) had 25-OHD deficiency (25–49 nmol/l), and 6 (6/36, 16.7%) had severe 25-OHD deficiency (25 nmol/l) (Table 4). Patients with limited ambulation due to AMC had lower 25-OHD levels (33.8 nmol/l vs*.* 53.5 nmol/l, p = 0.03) and were more likely to have severe 25-OHD deficiency (4/9, 44.4% vs. 2/27, 7.4%, p = 0.01). The blood 25-OHD level and the prevalence of 25-OHD insufficiency and deficiency did not differ between the Amyoplasia group and the other AMC group. No associations were detected between 25-OHD and lumbar, neck femoral or total hip BMD. Patients with severe vitamin D deficiency had poorer 6MWT performance (158 (± 164) m vs*.* 362 (± 204) m; p = 0.03), lower calcium levels (2.13 (± 0.17) mmol/l vs*.* 2.25 (± 0.09) mmol/l; p = 0.02), and more frequent hypocalcemia (50% (3/6) vs*.* 7.7% (2/24); p = 0.01).

**Table 4:** Blood 25-hydroxyvitamin D, calcium and phosphate levels in patients with arthrogryposis multiplex congenita.

	n	All patients with AMC	Amyoplasia	Other types of AMC	p value
25-OHD level (nmol/l) (mean (SD))	36	48.6 (24.2)	50.1 (23.7)	46.5 (25.5)	0.6
25-OHD < 75 nmol/l (n (%))		31 (86.1)	18 (85.7)	13 (86.7)	0.9
50 nmol/l) ≤ 25-OHD < 74 nmol/l (n (%))		10 (27.8)	7 (33.3)	3 (20.0)	0.5
25 nmol/l ≤ 25-OHD < 49 nmol/l (n (%))		15 (41.7)	8 (38.1)	7 (46.7)	0.7
25-OHD < 25 nmol/l (n (%))		6 (16.7)	3 (14.3)	3 (20.0)	0.7
Calcium level (mmol/l) (mean (SD))	33	2.23 (0.11)	2.24 (0.108)	2.21 (0.122)	0.4
Calcium < 2.12 mmol/l (n (%))		5 (15.2)	2 (10.5)	3 (21.4)	0.4
Calcium < 2.6 mmol/l (n (%))		0 (0)	0 (0)	0 (0)	ND
Phosphate level (mmol/l) (median [Q1-Q3])	32	0.98 [0.92–1.16]	1.00 [0.91–1.14]	0.97 [0.95–1.16] (0.17)	1.0
Phosphate < 0.80 mmol/l (n (%))		3 (9.4)	2 (11.1)	1 (7.1)	0.7
Phosphate > 1.45 mmol/l (n (%))		1 (3.1)	1 (5.6)	0 (0)	0.4

p values: Student’s *t* test, the Mann‒Whitney *U* test or the chi‒square test between the Amyoplasia group and the other types of AMC group. Normal 25-OHD level: 75–125 nmol/l; normal calcium level: 2.12–2.6 mmol/l; normal phosphate level: 0.80–1.60 mmol/l. Q1: first quartile, Q3: third quartile.

*AMC* Arthrogryposis multiplex congenita, *25-OHD* 25-hydroxyvitamin D, *ND* no data.

To determine whether the blood 25-OHD level differed between patients with AMC and healthy subjects, we compared the present results to those of a 2016 study that assessed blood 25-OHD levels in the general population in France^21^. AMC levels were lower in patients with AMC (48.5 ± 24.3 nmol/l) than in 30- to 59-year-old healthy subjects (60.8 ± 18.3 nmol/l); p = 0.006). Severe 25-OHD deficiency (25 nmol/l) was more frequent in patients with AMC (6/36, 16.7%) than in the healthy general population (6.3%; p = 0.03).

Five patients (5/33, 15.2%) had hypocalcemia (Table 3). All had low blood 25-OHD levels, and 60% (3/5) had severe 25-OHD deficiency. Calcium blood levels were significantly lower in limited ambulatory patients (2.15 ± 0.14 vs. 2.25 ± 0.09 mmol/l; p = 0.02).

## Discussion

The present study showed for the first time that adults with AMC had low femoral neck BMD and a higher frequency of vitamin D deficiency, whereas the BMD of the lumbar spine was not significantly impaired. Half of the adults with AMC reported a history of fracture during their lifetime.

A total of 14.3%, 22.5% and 25.0% of patients had lumbar spine, femoral neck and total hip BMD Z scores, respectively, below − 2, i.e. below the expected range for age as determined by the International Society for Clinical Densitometry. Hip BMD in patients with AMC was lower than expected in the healthy population, but this was not the case for the lumbar spine. The level of precision for BMD measurement of the lumbar spine, femoral neck, and total hip is excellent with Lunar iDXA^22^. The BMD values observed in patients with AMC cannot be explained by random measurement variability due to the instrument but indicate a true change in BMD. In our cohort, 30.4% of patients had a Z score less than − 2. This prevalence seems high and significant, especially when compared with the prevalence of low bone mass (defined as a Z score ≤  − 2) in other diseases that affect young people and are associated with an increased risk of fragility fractures, such as cystic fibrosis (52%), Cushing disease (44.5%), human immunodeficiency virus infection (35%) and systemic lupus erythematosus (17.3%)^23–27^.

Hall et al. have argued that fractures occur in patients with AMC not only because of the difficulties of delivery but also because of the BMD deficit associated with AMC^8^. In a study on a pediatric population with AMC, Spencer et al. reported that ambulatory function was weakly correlated with lumbar spine BMD. This observation suggest that, in this population, loading stimulation of the lower limb bones is not sufficient for effective bone remodeling, as also observed in patients with spinal cord injuries^28^. However in our study, patients with limited walking ability in the 6MWT did not have significantly lower BMD. Proteins encoded by *TNNI2*, *TRPV4* and *ZC4H2* are expressed by osteoclasts and osteoblasts and are involved in bone remodeling^29–31^. Germline mutations in these genes and their effects on protein expression and/or protein function may therefore at least partly explain the bone demineralization observed in patients with AMC linked to these genes. In addition, the fetal hypokinesia observed in patients with AMC may be responsible for the defect in BMD gain during this period, which persists into adulthood^6^.

DXA is the gold standard for measuring BMD to estimate future fracture risk^32^. Cohen et al. demonstrated that a low BMD defined by a Z score of − 2.0 or less in premenopausal women is associated with lower bone stiffness via voxel-based finite element analysis and alteration of the bone microarchitecture, even in the absence of a fragility fracture^33^. Therefore, having a BMD Z score of − 2 or less is likely to mean that the bone is structurally and biomechanically more fragile. The association between BMD on DXA and the risk of future fracture in patients with AMC, as observed in postmenopausal and premenopausal women^27,34,35^, needs to be investigated. In our cohort, half of the adults with AMC experienced a fracture in their lifetime, mainly at a peripheral location. Like Spencer et al*.* we did not observe an association between lumbar spine or hip BMD and a history of fracture^14^. However, the poor quality of the fracture data collected in this study and in that of Spencer et al. prevents us from drawing any firm conclusions^14^. In addition, the cross-sectional design of our study did not allow us to precisely assess the ability of DXA to predict fracture risk in the AMC.

Reliable measurement of BMD by DXA requires proper positioning of the lumbar spine and femoral necks, making this examination difficult to interpret in patients with structural skeletal abnormalities or in patients with limited joint range of motion^32^. Axial rotation of the vertebrae in patients with scoliosis leads to overestimation of the BMD beyond 5° of rotation^36^. A 10° internal rotation of the leg was reported to artificially increase femoral neck BMD, whereas the opposite effect was observed for external rotation^37^. In our study, 4 patients had severe scoliosis, making the BMD measurements uninterpretable. Eighteen femoral neck measurements were also considered uninterpretable due to incorrect patient positioning. However, all patients had at least one interpretable measurement, as we measured BMD at 3 sites (the lumbar spine, right hip and left hip), indicating that BMD measurement remains feasible in patients with AMC. Careful analysis of DXA results is essential to avoid drawing erroneous conclusions in adults with AMC. BMD measurements should preferably be performed on the hip with a normal range of motion to avoid difficulties in patient positioning, which can lead to measurement errors.

Vitamin D deficiency was observed more frequently in patients with AMC, particularly in patients with limited mobility, than in the general population. Furthermore, in this vitamin D-deficient population, 1 in 2 patients had hypocalcemia, indicating major impairment of phosphate and calcium metabolism, which is known to have a negative impact on bone health. Vitamin D is also important for muscle health. Vitamin D insufficiency is associated with the development of sarcopenia and accelerates the decline in physical performance^38,39^. Muscle weakness has been reported to occur in patients with AMC^11^. In the present study, patients with vitamin D deficiency exhibited weaker performance in the 6MWT. Screening for and correcting vitamin D deficiency, particularly in patients with walking disability, could be a health intervention with beneficial consequences for both bone and muscle function.

We believe that our study is among the largest bone densitometry studies on AMC, and it is the first to assess the prevalence of vitamin D insufficiency in adults with AMC.

However, our study had several limitations. The small sample size and the heterogeneity of the patients, due to the clinical definition of AMC and the rarity of this pathology, did not allow us to draw strong conclusions due to low statistical power. Not all patients underwent DXA analysis and vitamin D assays. In particular, vitamin D and calcium measurements were not systematically included in our screening protocol, which explains the missing data. We cannot exclude the possibility that the incidence of fractures was underestimated due to the recall bias inherent in the study design and the lack of systematic radiographic screening for vertebral fractures, although these fractures are often underdiagnosed. The proportion of fractures caused by low-level trauma equivalent to a fall from standing height or less could not be determined due to the lack of precise data in the medical records. We are therefore unable to estimate the prevalence of fragility fractures. Patients with severe spinal and hip contracture have difficulties positioning the spine and femoral neck as recommended, which can lead to inaccurate BMD measurements. We took this into account by asking two independent readers to assess the quality of the DXA scans. In our cohort, the prescription of vitamin D was not guided by prespecified instructions depending on the patient's pathology. Nevertheless, we observed that patients who received vitamin D had a lower weight. Therefore, we cannot exclude the possible existence of a selection bias in this analysis. Finally, to be included, patients had to visit the French National Center for AMC to obtain an expert evaluation, which could have induced a selection bias.

In conclusion, adults with AMC had lower hip BMD than expected for age and a higher frequency of vitamin D insufficiency. A history of fracture was common in this population. Screening for low BMD by DXA and specific interventions, such as vitamin D supplementation, should be considered in adults with AMC. DXA scans must be interpreted carefully, ensuring that the measurements are not influenced by scoliosis or difficulties in achieving the correct positioning, to avoid drawing erroneous conclusions.

### Supplementary Information

Supplementary Table S1.

Supplementary Table S2.

Supplementary Table S3.

Supplementary Table S4.

Supplementary Table S5.

## Data Availability

The data that support the findings of this study are available from the corresponding author upon reasonable request.
