# Supplementary material for: Bone mineral density in adults with arthrogryposis multiplex congenita: a retrospective cohort analysis
Source: Sci Rep. 2024 Apr 8;14:8206. doi: 10.1038/s41598-024-58083-x (PMC11001861; doi:10.1038/s41598-024-58083-x)
Supplement: Supplementary file 1 — Supplementary Table S1. [file 41598_2024_58083_MOESM1_ESM.docx]

**Table S1 : Comparaison between men and women**

| Factors | Men (n=17) | Women (n=34) | p-value |
| --- | --- | --- | --- |
| Age (years, mean (SD)) | 33.2 (14.4) | 32.8 (11.9) | 0.93 |
| Amyoplasia (n (%)) | 12 (70.6) | 20 (58.8) | 0.41 |
| Height (centimeters, mean (SD))^a^ | 158.7 (13.2) | 155.2 (7.9) | 0.28 |
| Weight (kilogram, mean (SD))^a^ | 54.5 (17.6) | 59.6 (17.0) | 0.36 |
| BMI (kg/m^2^, mean (SD))^a^ | 21.4 (5.9) | 24.9 (8.0) | 0.15 |
| History of fractures (n (%)) | 8 (47.1) | 17 (50.0) | 0.84 |
| Non ambulation (n (%)) | \| 6 \| (35.3) \| \| --- \| --- \| | 8 (23.5) | 0.38 |
| Total FIM (mean (SD)) | 112.3 (16.5) | 110.3 (15.6) | 0.67 |
| 6MWT (meters, mean (SD))^b^ | 321.4 (220.7) | 323.3 (203.5) | 0.98 |
| Calcium levels (mmol/l, mean (SD))^c^ | 2.3 (0.1) | 2.2 (0.1) | 0.31 |
| Phosphate levels (mmol/l, mean (SD))^d^ | 1.1 (0.3) | 1.0 (0.2) | 0.72 |
| 25-OHD levels (ng/ml, mean (SD)^e^ | 15.3 (8.6) | 21.2 (9.7) | 0.09 |
| Lumbar BMD (g/cm^2^, mean (SD))^f^ | 1.13 (0.23) | 1.14 (0.21) | 0.84 |
| Z-score lumbar BMD^g^ | 0.07 (1.6) | -0.08 (1.7) | 0.79 |
| Femoral neck BMD (g/cm^2^, mean (SD))^h^ | 0.89 (0.18) | 0.81 (0.14) | 0.13 |
| Z-score femoral neck BMD ^h^ | -0.69 (1.18) | -1.33 (1.03) | 0.09 |

SD : Standard Deviation, BMI : Body Mass Index, FIM : Functional Independence Measure, 6MWT : 6-minute walk test, 25-OHD : 25-hydroxyvitamin D, BMD : Bone Mineral Density, ^a^ missing data for 2 men and 3 women, ^b^ missing data for 1 men, ^c^ missing data for 6 men and 12 women, ^d^ missing data for 7 men and 12 women, ^e^ missing data for 6 men and 9 women, ^f^ missing data for 3 men and 5 women, ^g^  missing data for 4 men and 5 women, ^h^ missing data for 4 men and 7 women. p-value of Student’s t-test, chi-square test between men and women.
