# Supplementary material for: Bone mineral density in adults with arthrogryposis multiplex congenita: a retrospective cohort analysis
Source: Sci Rep. 2024 Apr 8;14:8206. doi: 10.1038/s41598-024-58083-x (PMC11001861; doi:10.1038/s41598-024-58083-x)
Supplement: Supplementary file 2 — Supplementary Table S2. [file 41598_2024_58083_MOESM2_ESM.docx]

| \|  \| \| \| \| \| \| \| \| \| \| \| \| \| \| \| \| \| \| \| \| \| \| \| \| \| --- \| --- \| --- \| --- \| --- \| --- \| --- \| --- \| --- \| --- \| --- \| --- \| --- \| --- \| --- \| --- \| --- \| --- \| --- \| --- \| --- \| --- \| --- \| --- \| \|  \| \|  \| \| **Lumbar BMD** \| \| **Height** \| \| **Weight** \| \| **BMI** \| \| **Age** \| \| **Calcium level** \| \| **Phosphate level** \| \| **25-OH level** \| \| **6MWT** \| \| **Total FIM** \| \| \| Lumbar BMD \|  \| rs \|  \| — \|  \|  \|  \|  \|  \|  \|  \|  \|  \|  \|  \|  \|  \|  \|  \|  \|  \|  \|  \| \|  \|  \| p value \|  \| — \|  \|  \|  \|  \|  \|  \|  \|  \|  \|  \|  \|  \|  \|  \|  \|  \|  \|  \|  \| \|  \|  \| N \|  \| — \|  \|  \|  \|  \|  \|  \|  \|  \|  \|  \|  \|  \|  \|  \|  \|  \|  \|  \|  \| \| Height \|  \| rs \|  \| 0.362 \| * \| — \|  \|  \|  \|  \|  \|  \|  \|  \|  \|  \|  \|  \|  \|  \|  \|  \|  \| \|  \|  \| p value \|  \| 0.019 \|  \| — \|  \|  \|  \|  \|  \|  \|  \|  \|  \|  \|  \|  \|  \|  \|  \|  \|  \| \|  \|  \| N \|  \| 42 \|  \| — \|  \|  \|  \|  \|  \|  \|  \|  \|  \|  \|  \|  \|  \|  \|  \|  \|  \| \| Weight \|  \| rs \|  \| 0.371 \| * \| 0.207 \|  \| — \|  \|  \|  \|  \|  \|  \|  \|  \|  \|  \|  \|  \|  \|  \|  \| \|  \|  \| p value \|  \| 0.016 \|  \| 0.162 \|  \| — \|  \|  \|  \|  \|  \|  \|  \|  \|  \|  \|  \|  \|  \|  \|  \| \|  \|  \| N \|  \| 42 \|  \| 47 \|  \| — \|  \|  \|  \|  \|  \|  \|  \|  \|  \|  \|  \|  \|  \|  \|  \| \| BMI \|  \| rs \|  \| 0.331 \| * \| -0.186 \|  \| 0.888 \| *** \| — \|  \|  \|  \|  \|  \|  \|  \|  \|  \|  \|  \|  \|  \| \|  \|  \| p value \|  \| 0.032 \|  \| 0.215 \|  \| < .001 \|  \| — \|  \|  \|  \|  \|  \|  \|  \|  \|  \|  \|  \|  \|  \| \|  \|  \| N \|  \| 42 \|  \| 46 \|  \| 46 \|  \| — \|  \|  \|  \|  \|  \|  \|  \|  \|  \|  \|  \|  \|  \| \| Age \|  \| rs \|  \| 0.138 \|  \| 0.028 \|  \| 0.252 \|  \| 0.244 \|  \| — \|  \|  \|  \|  \|  \|  \|  \|  \|  \|  \|  \| \|  \|  \| p value \|  \| 0.384 \|  \| 0.854 \|  \| 0.088 \|  \| 0.102 \|  \| — \|  \|  \|  \|  \|  \|  \|  \|  \|  \|  \|  \| \|  \|  \| N \|  \| 42 \|  \| 47 \|  \| 47 \|  \| 46 \|  \| — \|  \|  \|  \|  \|  \|  \|  \|  \|  \|  \|  \| \| Calcium level \|  \| rs \|  \| 0.221 \|  \| 0.241 \|  \| -0.081 \|  \| -0.056 \|  \| -0.432 \| * \| — \|  \|  \|  \|  \|  \|  \|  \|  \|  \| \|  \|  \| p value \|  \| 0.278 \|  \| 0.209 \|  \| 0.677 \|  \| 0.772 \|  \| 0.012 \|  \| — \|  \|  \|  \|  \|  \|  \|  \|  \|  \| \|  \|  \| N \|  \| 26 \|  \| 29 \|  \| 29 \|  \| 29 \|  \| 33 \|  \| — \|  \|  \|  \|  \|  \|  \|  \|  \|  \| \| Phosphate level \|  \| rs \|  \| 0.052 \|  \| -0.154 \|  \| -0.029 \|  \| 0.085 \|  \| -0.226 \|  \| -0.023 \|  \| — \|  \|  \|  \|  \|  \|  \|  \| \|  \|  \| p value \|  \| 0.805 \|  \| 0.432 \|  \| 0.885 \|  \| 0.667 \|  \| 0.213 \|  \| 0.901 \|  \| — \|  \|  \|  \|  \|  \|  \|  \| \|  \|  \| N \|  \| 25 \|  \| 28 \|  \| 28 \|  \| 28 \|  \| 32 \|  \| 32 \|  \| — \|  \|  \|  \|  \|  \|  \|  \| \| 25-OHD level \|  \| rs \|  \| -0.162 \|  \| 0.265 \|  \| -0.151 \|  \| -0.231 \|  \| -0.065 \|  \| 0.226 \|  \| -0.191 \|  \| — \|  \|  \|  \|  \|  \| \|  \|  \| p value \|  \| 0.409 \|  \| 0.149 \|  \| 0.418 \|  \| 0.210 \|  \| 0.706 \|  \| 0.215 \|  \| 0.304 \|  \| — \|  \|  \|  \|  \|  \| \|  \|  \| N \|  \| 28 \|  \| 31 \|  \| 31 \|  \| 31 \|  \| 36 \|  \| 32 \|  \| 31 \|  \| — \|  \|  \|  \|  \|  \| \| 6MWT \|  \| rs \|  \| 0.165 \|  \| 0.629 \| *** \| 0.002 \|  \| -0.236 \|  \| -0.154 \|  \| 0.367 \| * \| -0.395 \| * \| 0.296 \|  \| — \|  \|  \|  \| \|  \|  \| p value \|  \| 0.302 \|  \| < .001 \|  \| 0.989 \|  \| 0.118 \|  \| 0.262 \|  \| 0.039 \|  \| 0.028 \|  \| 0.084 \|  \| — \|  \|  \|  \| \|  \|  \| N \|  \| 41 \|  \| 46 \|  \| 46 \|  \| 45 \|  \| 55 \|  \| 32 \|  \| 31 \|  \| 35 \|  \| — \|  \|  \|  \| \| Total FIM \|  \| rs \|  \| 0.079 \|  \| 0.408 \| ** \| -0.175 \|  \| -0.307 \| * \| -0.098 \|  \| 0.350 \| * \| -0.223 \|  \| 0.135 \|  \| 0.764 \| *** \| — \|  \| \|  \|  \| p value \|  \| 0.619 \|  \| 0.004 \|  \| 0.239 \|  \| 0.038 \|  \| 0.472 \|  \| 0.046 \|  \| 0.219 \|  \| 0.432 \|  \| < .001 \|  \| — \|  \| \|  \|  \| N \|  \| 42 \|  \| 47 \|  \| 47 \|  \| 46 \|  \| 56 \|  \| 33 \|  \| 32 \|  \| 36 \|  \| 55 \|  \| — \|  \| \| **Tableau S2: Correlation matrix for lumbar Bone Mineral Density.**  BMD: Bone Mineral Density, BMI : Body Mass Index, 25-OHD : 25-hydroxyvitamin D, 6MWT : 6 minutes walk test, FIM : functional independence measure, rs : Spearman's rank correlation coefficient. * p < .05, ** p < .01, *** p < .001 \| \| \| \| \| \| \| \| \| \| \| \| \| \| \| \| \| \| \| \| \| \| \| \| \|  \| \| \| \| \| \| \| \| \| \| \| \| \| \| \| \| \| \| \| \| \| \| \| \| |  |
| --- | --- | --- | --- | --- | --- | --- | --- | --- | --- | --- | --- | --- | --- | --- | --- | --- | --- | --- | --- | --- | --- | --- | --- | --- | --- | --- | --- | --- | --- | --- | --- | --- | --- | --- | --- | --- | --- | --- | --- | --- | --- | --- | --- | --- | --- | --- | --- | --- | --- | --- | --- | --- | --- | --- | --- | --- | --- | --- | --- | --- | --- | --- | --- | --- | --- | --- | --- | --- | --- | --- | --- | --- | --- | --- | --- | --- | --- | --- | --- | --- | --- | --- | --- | --- | --- | --- | --- | --- | --- | --- | --- | --- | --- | --- | --- | --- | --- | --- | --- | --- | --- | --- | --- | --- | --- | --- | --- | --- | --- | --- | --- | --- | --- | --- | --- | --- | --- | --- | --- | --- | --- | --- | --- | --- | --- | --- | --- | --- | --- | --- | --- | --- | --- | --- | --- | --- | --- | --- | --- | --- | --- | --- | --- | --- | --- | --- | --- | --- | --- | --- | --- | --- | --- | --- | --- | --- | --- | --- | --- | --- | --- | --- | --- | --- | --- | --- | --- | --- | --- | --- | --- | --- | --- | --- | --- | --- | --- | --- | --- | --- | --- | --- | --- | --- | --- | --- | --- | --- | --- | --- | --- | --- | --- | --- | --- | --- | --- | --- | --- | --- | --- | --- | --- | --- | --- | --- | --- | --- | --- | --- | --- | --- | --- | --- | --- | --- | --- | --- | --- | --- | --- | --- | --- | --- | --- | --- | --- | --- | --- | --- | --- | --- | --- | --- | --- | --- | --- | --- | --- | --- | --- | --- | --- | --- | --- | --- | --- | --- | --- | --- | --- | --- | --- | --- | --- | --- | --- | --- | --- | --- | --- | --- | --- | --- | --- | --- | --- | --- | --- | --- | --- | --- | --- | --- | --- | --- | --- | --- | --- | --- | --- | --- | --- | --- | --- | --- | --- | --- | --- | --- | --- | --- | --- | --- | --- | --- | --- | --- | --- | --- | --- | --- | --- | --- | --- | --- | --- | --- | --- | --- | --- | --- | --- | --- | --- | --- | --- | --- | --- | --- | --- | --- | --- | --- | --- | --- | --- | --- | --- | --- | --- | --- | --- | --- | --- | --- | --- | --- | --- | --- | --- | --- | --- | --- | --- | --- | --- | --- | --- | --- | --- | --- | --- | --- | --- | --- | --- | --- | --- | --- | --- | --- | --- | --- | --- | --- | --- | --- | --- | --- | --- | --- | --- | --- | --- | --- | --- | --- | --- | --- | --- | --- | --- | --- | --- | --- | --- | --- | --- | --- | --- | --- | --- | --- | --- | --- | --- | --- | --- | --- | --- | --- | --- | --- | --- | --- | --- | --- | --- | --- | --- | --- | --- | --- | --- | --- | --- | --- | --- | --- | --- | --- | --- | --- | --- | --- | --- | --- | --- | --- | --- | --- | --- | --- | --- | --- | --- | --- | --- | --- | --- | --- | --- | --- | --- | --- | --- | --- | --- | --- | --- | --- | --- | --- | --- | --- | --- | --- | --- | --- | --- | --- | --- | --- | --- | --- | --- | --- | --- | --- | --- | --- | --- | --- | --- | --- | --- | --- | --- | --- | --- | --- | --- | --- | --- | --- | --- | --- | --- | --- | --- | --- | --- | --- | --- | --- | --- | --- | --- | --- | --- | --- | --- | --- | --- | --- | --- | --- | --- | --- | --- | --- | --- | --- | --- | --- | --- | --- | --- | --- | --- | --- | --- | --- | --- | --- | --- | --- | --- | --- | --- | --- | --- | --- | --- | --- | --- | --- | --- | --- | --- | --- | --- | --- | --- | --- | --- | --- | --- | --- | --- | --- | --- | --- | --- | --- | --- | --- | --- | --- | --- | --- | --- | --- | --- | --- | --- | --- | --- | --- | --- | --- | --- | --- | --- | --- | --- | --- | --- | --- | --- | --- | --- | --- | --- | --- | --- | --- | --- | --- | --- | --- | --- | --- | --- | --- | --- | --- | --- | --- | --- | --- | --- | --- | --- | --- | --- | --- | --- | --- | --- | --- | --- | --- | --- | --- | --- | --- | --- | --- | --- | --- | --- | --- | --- | --- | --- | --- | --- | --- | --- | --- | --- | --- | --- | --- | --- | --- | --- | --- | --- | --- | --- | --- | --- | --- | --- | --- | --- | --- | --- | --- | --- | --- | --- | --- | --- | --- | --- | --- | --- | --- | --- | --- | --- | --- | --- | --- | --- | --- | --- | --- | --- | --- | --- | --- | --- | --- | --- | --- | --- | --- | --- | --- | --- | --- | --- | --- | --- | --- | --- | --- | --- | --- | --- | --- | --- | --- | --- | --- | --- | --- | --- | --- | --- | --- | --- | --- | --- | --- | --- | --- | --- | --- | --- | --- | --- | --- | --- | --- | --- | --- | --- | --- | --- | --- | --- | --- | --- | --- | --- | --- | --- | --- | --- | --- | --- | --- | --- | --- | --- | --- | --- | --- | --- | --- | --- | --- | --- | --- | --- | --- | --- | --- | --- | --- | --- | --- | --- | --- | --- | --- | --- | --- | --- | --- | --- | --- | --- | --- | --- | --- | --- | --- | --- | --- | --- | --- | --- | --- | --- | --- | --- | --- | --- | --- | --- | --- | --- | --- | --- | --- | --- | --- | --- | --- | --- | --- | --- | --- | --- | --- | --- | --- | --- | --- | --- | --- | --- | --- | --- | --- | --- | --- | --- | --- | --- |
|  | |
|  | |
