# Supplementary material for: Bone mineral density in adults with arthrogryposis multiplex congenita: a retrospective cohort analysis
Source: Sci Rep. 2024 Apr 8;14:8206. doi: 10.1038/s41598-024-58083-x (PMC11001861; doi:10.1038/s41598-024-58083-x)
Supplement: Supplementary file 3 — Supplementary Table S3. [file 41598_2024_58083_MOESM3_ESM.docx]

|  | | | | | | | | | | | | | | | | | | | | | | | |
| --- | --- | --- | --- | --- | --- | --- | --- | --- | --- | --- | --- | --- | --- | --- | --- | --- | --- | --- | --- | --- | --- | --- | --- |
|  | |  | | **Femoral neck BMD** | | **Height** | | **Weight** | | **BMI** | | **Age** | | **Calcium level** | | **Phosphate level** | | **25-OHD level** | | **6MWT** | | **Total FIM** | |
| Femoral neck BMD |  | rs |  | — |  | 0.393 | * | 0.255 |  | 0.221 |  | -0.315 | * | 0.395 |  | 0.313 |  | -0.023 |  | 0.300 |  | 0.228 |  |
|  |  | p value |  | — |  | 0.012 |  | 0.112 |  | 0.170 |  | 0.048 |  | 0.051 |  | 0.136 |  | 0.909 |  | 0.063 |  | 0.156 |  |
|  |  | N |  | — |  | 40 |  | 40 |  | 40 |  | 40 |  | 25 |  | 24 |  | 27 |  | 39 |  | 40 |  |
| Height |  | rs |  |  |  | — |  |  |  |  |  |  |  |  |  |  |  |  |  |  |  |  |  |
|  |  | p value |  |  |  | — |  |  |  |  |  |  |  |  |  |  |  |  |  |  |  |  |  |
|  |  | N |  |  |  | — |  |  |  |  |  |  |  |  |  |  |  |  |  |  |  |  |  |
| Weigth |  | rs |  |  |  | 0.207 |  | — |  |  |  |  |  |  |  |  |  |  |  |  |  |  |  |
|  |  | p value |  |  |  | 0.162 |  | — |  |  |  |  |  |  |  |  |  |  |  |  |  |  |  |
|  |  | N |  |  |  | 47 |  | — |  |  |  |  |  |  |  |  |  |  |  |  |  |  |  |
| BMI |  | rs |  |  |  | -0.186 |  | 0.888 | *** | — |  |  |  |  |  |  |  |  |  |  |  |  |  |
|  |  | p value |  |  |  | 0.215 |  | < .001 |  | — |  |  |  |  |  |  |  |  |  |  |  |  |  |
|  |  | N |  |  |  | 46 |  | 46 |  | — |  |  |  |  |  |  |  |  |  |  |  |  |  |
| Age |  | rs |  |  |  | 0.028 |  | 0.252 |  | 0.244 |  | — |  |  |  |  |  |  |  |  |  |  |  |
|  |  | p value |  |  |  | 0.854 |  | 0.088 |  | 0.102 |  | — |  |  |  |  |  |  |  |  |  |  |  |
|  |  | N |  |  |  | 47 |  | 47 |  | 46 |  | — |  |  |  |  |  |  |  |  |  |  |  |
| Calcium level |  | rs |  |  |  | 0.241 |  | -0.081 |  | -0.056 |  | -0.432 | * | — |  |  |  |  |  |  |  |  |  |
|  |  | p value |  |  |  | 0.209 |  | 0.677 |  | 0.772 |  | 0.012 |  | — |  |  |  |  |  |  |  |  |  |
|  |  | N |  |  |  | 29 |  | 29 |  | 29 |  | 33 |  | — |  |  |  |  |  |  |  |  |  |
| Phosphate level |  | rs |  |  |  | -0.154 |  | -0.029 |  | 0.085 |  | -0.226 |  | -0.023 |  | — |  |  |  |  |  |  |  |
|  |  | p value |  |  |  | 0.432 |  | 0.885 |  | 0.667 |  | 0.213 |  | 0.901 |  | — |  |  |  |  |  |  |  |
|  |  | N |  |  |  | 28 |  | 28 |  | 28 |  | 32 |  | 32 |  | — |  |  |  |  |  |  |  |
| 25-OHD level |  | rs |  |  |  | 0.265 |  | -0.151 |  | -0.231 |  | -0.065 |  | 0.226 |  | -0.191 |  | — |  |  |  |  |  |
|  |  | p value |  |  |  | 0.149 |  | 0.418 |  | 0.210 |  | 0.706 |  | 0.215 |  | 0.304 |  | — |  |  |  |  |  |
|  |  | N |  |  |  | 31 |  | 31 |  | 31 |  | 36 |  | 32 |  | 31 |  | — |  |  |  |  |  |
| TEST DE 6 MIN |  | rs |  |  |  | 0.629 | *** | 0.002 |  | -0.236 |  | -0.154 |  | 0.367 | * | -0.395 | * | 0.296 |  | — |  |  |  |
|  |  | p value |  |  |  | < .001 |  | 0.989 |  | 0.118 |  | 0.262 |  | 0.039 |  | 0.028 |  | 0.084 |  | — |  |  |  |
|  |  | N |  |  |  | 46 |  | 46 |  | 45 |  | 55 |  | 32 |  | 31 |  | 35 |  | — |  |  |  |
| MIF totale |  | rs |  |  |  | 0.408 | ** | -0.175 |  | -0.307 | * | -0.098 |  | 0.350 | * | -0.223 |  | 0.135 |  | 0.764 | *** | — |  |
|  |  | p value |  |  |  | 0.004 |  | 0.239 |  | 0.038 |  | 0.472 |  | 0.046 |  | 0.219 |  | 0.432 |  | < .001 |  | — |  |
|  |  | N |  |  |  | 47 |  | 47 |  | 46 |  | 56 |  | 33 |  | 32 |  | 36 |  | 55 |  | — |  |
|  | | | | | | | | | | | | | | | | | | | | | | | |
|  | | | | | | | | | | | | | | | | | | | | | | | |

**Tableau S3: Correlation matrix for femoral neck Bone Mineral Density.**

BMD: Bone Mineral Density, BMI: Body Mass Index, 25-OHD: 25-hydroxyvitamin D, 6MWT: 6 minute walk test, FIM : functional independence measure, * p < .05, ** p < .01, *** p < .001
