# Supplementary material for: Bone mineral density in adults with arthrogryposis multiplex congenita: a retrospective cohort analysis
Source: Sci Rep. 2024 Apr 8;14:8206. doi: 10.1038/s41598-024-58083-x (PMC11001861; doi:10.1038/s41598-024-58083-x)
Supplement: Supplementary file 4 — Supplementary Table S4. [file 41598_2024_58083_MOESM4_ESM.docx]

| \|  \| \| \| \| \| \| \| \| \| \| \| \| \| \| \| \| \| \| \| \| \| \| \| \| \| \| \| \| --- \| --- \| --- \| --- \| --- \| --- \| --- \| --- \| --- \| --- \| --- \| --- \| --- \| --- \| --- \| --- \| --- \| --- \| --- \| --- \| --- \| --- \| --- \| --- \| --- \| --- \| --- \| \|  \| \|  \| \| **Total hip BMD** \| \| \| **Height** \| \| **Weight** \| \| **BMI** \| \| **Age** \| \| **Calcium level** \| \| **Phosphate level** \| \| **25-OHD level** \| \| **6MWT** \| \| \| **Total FIM** \| \| \| \| Total hip BMD \|  \| rs \|  \| \| — \|  \|  \|  \|  \|  \|  \|  \|  \|  \|  \|  \|  \|  \|  \|  \|  \|  \|  \| \|  \| \|  \|  \| p value \|  \| \| — \|  \|  \|  \|  \|  \|  \|  \|  \|  \|  \|  \|  \|  \|  \|  \|  \|  \|  \| \|  \| \|  \|  \| N \|  \| \| — \|  \|  \|  \|  \|  \|  \|  \|  \|  \|  \|  \|  \|  \|  \|  \|  \|  \|  \| \|  \| \| Height \|  \| rs \|  \| \| 0.331 \| * \| — \|  \|  \|  \|  \|  \|  \|  \|  \|  \|  \|  \|  \|  \|  \|  \|  \| \|  \| \|  \|  \| p value \|  \| \| 0.037 \|  \| — \|  \|  \|  \|  \|  \|  \|  \|  \|  \|  \|  \|  \|  \|  \|  \|  \| \|  \| \|  \|  \| N \|  \| \| 40 \|  \| — \|  \|  \|  \|  \|  \|  \|  \|  \|  \|  \|  \|  \|  \|  \|  \|  \| \|  \| \| Weight \|  \| rs \|  \| \| 0.198 \|  \| 0.207 \|  \| — \|  \|  \|  \|  \|  \|  \|  \|  \|  \|  \|  \|  \|  \|  \| \|  \| \|  \|  \| p value \|  \| \| 0.221 \|  \| 0.162 \|  \| — \|  \|  \|  \|  \|  \|  \|  \|  \|  \|  \|  \|  \|  \|  \| \|  \| \|  \|  \| N \|  \| \| 40 \|  \| 47 \|  \| — \|  \|  \|  \|  \|  \|  \|  \|  \|  \|  \|  \|  \|  \|  \| \|  \| \| BMI \|  \| rs \|  \| \| 0.162 \|  \| -0.186 \|  \| 0.888 \| *** \| — \|  \|  \|  \|  \|  \|  \|  \|  \|  \|  \|  \|  \| \|  \| \|  \|  \| p value \|  \| \| 0.318 \|  \| 0.215 \|  \| < .001 \|  \| — \|  \|  \|  \|  \|  \|  \|  \|  \|  \|  \|  \|  \| \|  \| \|  \|  \| N \|  \| \| 40 \|  \| 46 \|  \| 46 \|  \| — \|  \|  \|  \|  \|  \|  \|  \|  \|  \|  \|  \|  \| \|  \| \| Age \|  \| rs \|  \| \| -0.175 \|  \| 0.028 \|  \| 0.252 \|  \| 0.244 \|  \| — \|  \|  \|  \|  \|  \|  \|  \|  \|  \|  \| \|  \| \|  \|  \| p value \|  \| \| 0.279 \|  \| 0.854 \|  \| 0.088 \|  \| 0.102 \|  \| — \|  \|  \|  \|  \|  \|  \|  \|  \|  \|  \| \|  \| \|  \|  \| N \|  \| \| 40 \|  \| 47 \|  \| 47 \|  \| 46 \|  \| — \|  \|  \|  \|  \|  \|  \|  \|  \|  \|  \| \|  \| \| Calcium level \|  \| rs \|  \| \| 0.411 \| * \| 0.241 \|  \| -0.081 \|  \| -0.056 \|  \| -0.432 \| * \| — \|  \|  \|  \|  \|  \|  \|  \|  \| \|  \| \|  \|  \| p value \|  \| \| 0.042 \|  \| 0.209 \|  \| 0.677 \|  \| 0.772 \|  \| 0.012 \|  \| — \|  \|  \|  \|  \|  \|  \|  \|  \| \|  \| \|  \|  \| N \|  \| \| 25 \|  \| 29 \|  \| 29 \|  \| 29 \|  \| 33 \|  \| — \|  \|  \|  \|  \|  \|  \|  \|  \| \|  \| \| Phosphate level \|  \| rs \|  \| \| 0.148 \|  \| -0.154 \|  \| -0.029 \|  \| 0.085 \|  \| -0.226 \|  \| -0.023 \|  \| — \|  \|  \|  \|  \|  \|  \| \|  \| \|  \|  \| p value \|  \| \| 0.490 \|  \| 0.432 \|  \| 0.885 \|  \| 0.667 \|  \| 0.213 \|  \| 0.901 \|  \| — \|  \|  \|  \|  \|  \|  \| \|  \| \|  \|  \| N \|  \| \| 24 \|  \| 28 \|  \| 28 \|  \| 28 \|  \| 32 \|  \| 32 \|  \| — \|  \|  \|  \|  \|  \|  \| \|  \| \| 25-OHD level \|  \| rs \|  \| \| -0.123 \|  \| 0.265 \|  \| -0.151 \|  \| -0.231 \|  \| -0.065 \|  \| 0.226 \|  \| -0.191 \|  \| — \|  \|  \|  \|  \| \|  \| \|  \|  \| p value \|  \| \| 0.542 \|  \| 0.149 \|  \| 0.418 \|  \| 0.210 \|  \| 0.706 \|  \| 0.215 \|  \| 0.304 \|  \| — \|  \|  \|  \|  \| \|  \| \|  \|  \| N \|  \| \| 27 \|  \| 31 \|  \| 31 \|  \| 31 \|  \| 36 \|  \| 32 \|  \| 31 \|  \| — \|  \|  \|  \|  \| \|  \| \| 6MWT \|  \| rs \|  \| \| 0.200 \|  \| 0.629 \| *** \| 0.002 \|  \| -0.236 \|  \| -0.154 \|  \| 0.367 \| * \| -0.395 \| * \| 0.296 \|  \| — \|  \|  \| \|  \| \|  \|  \| p value \|  \| \| 0.222 \|  \| < .001 \|  \| 0.989 \|  \| 0.118 \|  \| 0.262 \|  \| 0.039 \|  \| 0.028 \|  \| 0.084 \|  \| — \|  \|  \| \|  \| \|  \|  \| N \|  \| \| 39 \|  \| 46 \|  \| 46 \|  \| 45 \|  \| 55 \|  \| 32 \|  \| 31 \|  \| 35 \|  \| — \|  \|  \| \|  \| \| Total FIM \|  \| rs \|  \| \| 0.245 \|  \| 0.408 \| ** \| -0.175 \|  \| -0.307 \| * \| -0.098 \|  \| 0.350 \| * \| -0.223 \|  \| 0.135 \|  \| 0.764 \| *** \| — \| \|  \| \|  \|  \| p value \|  \| \| 0.128 \|  \| 0.004 \|  \| 0.239 \|  \| 0.038 \|  \| 0.472 \|  \| 0.046 \|  \| 0.219 \|  \| 0.432 \|  \| < .001 \|  \| — \| \|  \| \|  \|  \| N \|  \| \| 40 \|  \| 47 \|  \| 47 \|  \| 46 \|  \| 56 \|  \| 33 \|  \| 32 \|  \| 36 \|  \| 55 \|  \| — \| \|  \| |
| --- | --- | --- | --- | --- | --- | --- | --- | --- | --- | --- | --- | --- | --- | --- | --- | --- | --- | --- | --- | --- | --- | --- | --- | --- | --- | --- | --- | --- | --- | --- | --- | --- | --- | --- | --- | --- | --- | --- | --- | --- | --- | --- | --- | --- | --- | --- | --- | --- | --- | --- | --- | --- | --- | --- | --- | --- | --- | --- | --- | --- | --- | --- | --- | --- | --- | --- | --- | --- | --- | --- | --- | --- | --- | --- | --- | --- | --- | --- | --- | --- | --- | --- | --- | --- | --- | --- | --- | --- | --- | --- | --- | --- | --- | --- | --- | --- | --- | --- | --- | --- | --- | --- | --- | --- | --- | --- | --- | --- | --- | --- | --- | --- | --- | --- | --- | --- | --- | --- | --- | --- | --- | --- | --- | --- | --- | --- | --- | --- | --- | --- | --- | --- | --- | --- | --- | --- | --- | --- | --- | --- | --- | --- | --- | --- | --- | --- | --- | --- | --- | --- | --- | --- | --- | --- | --- | --- | --- | --- | --- | --- | --- | --- | --- | --- | --- | --- | --- | --- | --- | --- | --- | --- | --- | --- | --- | --- | --- | --- | --- | --- | --- | --- | --- | --- | --- | --- | --- | --- | --- | --- | --- | --- | --- | --- | --- | --- | --- | --- | --- | --- | --- | --- | --- | --- | --- | --- | --- | --- | --- | --- | --- | --- | --- | --- | --- | --- | --- | --- | --- | --- | --- | --- | --- | --- | --- | --- | --- | --- | --- | --- | --- | --- | --- | --- | --- | --- | --- | --- | --- | --- | --- | --- | --- | --- | --- | --- | --- | --- | --- | --- | --- | --- | --- | --- | --- | --- | --- | --- | --- | --- | --- | --- | --- | --- | --- | --- | --- | --- | --- | --- | --- | --- | --- | --- | --- | --- | --- | --- | --- | --- | --- | --- | --- | --- | --- | --- | --- | --- | --- | --- | --- | --- | --- | --- | --- | --- | --- | --- | --- | --- | --- | --- | --- | --- | --- | --- | --- | --- | --- | --- | --- | --- | --- | --- | --- | --- | --- | --- | --- | --- | --- | --- | --- | --- | --- | --- | --- | --- | --- | --- | --- | --- | --- | --- | --- | --- | --- | --- | --- | --- | --- | --- | --- | --- | --- | --- | --- | --- | --- | --- | --- | --- | --- | --- | --- | --- | --- | --- | --- | --- | --- | --- | --- | --- | --- | --- | --- | --- | --- | --- | --- | --- | --- | --- | --- | --- | --- | --- | --- | --- | --- | --- | --- | --- | --- | --- | --- | --- | --- | --- | --- | --- | --- | --- | --- | --- | --- | --- | --- | --- | --- | --- | --- | --- | --- | --- | --- | --- | --- | --- | --- | --- | --- | --- | --- | --- | --- | --- | --- | --- | --- | --- | --- | --- | --- | --- | --- | --- | --- | --- | --- | --- | --- | --- | --- | --- | --- | --- | --- | --- | --- | --- | --- | --- | --- | --- | --- | --- | --- | --- | --- | --- | --- | --- | --- | --- | --- | --- | --- | --- | --- | --- | --- | --- | --- | --- | --- | --- | --- | --- | --- | --- | --- | --- | --- | --- | --- | --- | --- | --- | --- | --- | --- | --- | --- | --- | --- | --- | --- | --- | --- | --- | --- | --- | --- | --- | --- | --- | --- | --- | --- | --- | --- | --- | --- | --- | --- | --- | --- | --- | --- | --- | --- | --- | --- | --- | --- | --- | --- | --- | --- | --- | --- | --- | --- | --- | --- | --- | --- | --- | --- | --- | --- | --- | --- | --- | --- | --- | --- | --- | --- | --- | --- | --- | --- | --- | --- | --- | --- | --- | --- | --- | --- | --- | --- | --- | --- | --- | --- | --- | --- | --- | --- | --- | --- | --- | --- | --- | --- | --- | --- | --- | --- | --- | --- | --- | --- | --- | --- | --- | --- | --- | --- | --- | --- | --- | --- | --- | --- | --- | --- | --- | --- | --- | --- | --- | --- | --- | --- | --- | --- | --- | --- | --- | --- | --- | --- | --- | --- | --- | --- | --- | --- | --- | --- | --- | --- | --- | --- | --- | --- | --- | --- | --- | --- | --- | --- | --- | --- | --- | --- | --- | --- | --- | --- | --- | --- | --- | --- | --- | --- | --- | --- | --- | --- | --- | --- | --- | --- | --- | --- | --- | --- | --- | --- | --- | --- | --- | --- | --- | --- | --- | --- | --- | --- | --- | --- | --- | --- | --- | --- | --- | --- | --- | --- | --- | --- | --- | --- | --- | --- | --- | --- | --- | --- | --- | --- | --- | --- | --- | --- | --- | --- | --- | --- | --- | --- | --- | --- | --- | --- | --- | --- | --- | --- | --- | --- | --- | --- | --- | --- | --- | --- | --- | --- | --- | --- | --- | --- | --- | --- | --- | --- | --- | --- | --- | --- | --- | --- | --- | --- | --- | --- | --- | --- | --- | --- | --- | --- | --- | --- | --- | --- | --- | --- | --- | --- | --- | --- | --- | --- | --- | --- | --- | --- | --- | --- | --- | --- | --- | --- | --- | --- | --- | --- | --- | --- | --- | --- | --- | --- | --- | --- | --- | --- | --- | --- | --- | --- | --- | --- | --- | --- | --- | --- | --- | --- | --- | --- | --- | --- | --- | --- | --- | --- | --- | --- | --- | --- | --- | --- | --- | --- | --- | --- | --- | --- | --- | --- | --- | --- | --- | --- | --- | --- | --- | --- | --- | --- | --- | --- | --- | --- | --- | --- | --- | --- | --- | --- | --- | --- | --- | --- | --- |
| **Tableau S4: Correlation matrix for total hip Bone Mineral Density.**  BMD: Bone Mineral Density, BMI: Body Mass Index, 25-OHD: 25-hydroxyvitamin D, 6MWT : 6 minute walk test, FIM: functional independence measure, * p < .05, ** p < .01, *** p < .001 |
|  |
