# Supplementary material for: Bone mineral density in adults with arthrogryposis multiplex congenita: a retrospective cohort analysis
Source: Sci Rep. 2024 Apr 8;14:8206. doi: 10.1038/s41598-024-58083-x (PMC11001861; doi:10.1038/s41598-024-58083-x)
Supplement: Supplementary file 5 — Supplementary Table S5. [file 41598_2024_58083_MOESM5_ESM.docx]

| Abbreviations | Definition |
| --- | --- |
| 6MWT | 6 Minutes walk test |
| AMC | Arthrogryposis Multiplex Congenita |
| BMD | Bone Mineral Density |
| BMI | Body Mass Index |
| DXA | Dual-energy X-ray Absorptiometry |
| FIM | Functional Independence Measure |
| ISCD | International Society of Clinical Densitometry |
| OHD | hydroxyvitamin D |
| PARART | Pediatric and Adult Registry for patients with ARThrogryposis multiplex congenita |

Table S5. List of abbreviations
